# Clinical-epidemiological profile of leprosy patients undergoing retreatment at a reference center in southeastern Brazil^☆^

**DOI:** 10.1016/j.abd.2023.09.006

**Published:** 2024-05-15

**Authors:** Guilherme Holanda Bezerra, Paulo Eduardo Neves Ferreira Velho, Andréa Fernandes Eloy da Costa França

**Affiliations:** a Clinical Specialties Unit, Hospital das Clínicas, Universidade Federal de Goiás, Goiânia, GO, Brazil; b Department of Dermatology, Universidade Estadual de Campinas, Campinas, SP, Brazil

Dear Editor,

Leprosy is a chronic infectious disease caused by acid-fast bacilli of the *Mycobacterium leprae* complex, with a predilection for peripheral nerves and skin. The high incidence and morbidity of the disease in Brazil classifies it as a public health problem.^1^, ^2^ Leprosy diagnosis is mainly clinical-epidemiological and can be complemented by direct screening of the agent, laboratory and histopathology.^3^

Treatment varies according to the operational classification as paucibacillary (PB) or multibacillary (MB), with the combination of rifampicin, dapsone, and clofazimine (multidrug therapy or MDT), for six to twelve months.^1^

Cases that require retreatment may be classified as recurrence, therapeutic insufficiency, therapeutic failure, or reinfection, based on clinical and laboratory criteria. In official documents, leprosy recurrence occurs in cases regularly treated with correctly indicated regimens that present clinical disease after discharge due to cure. Therapeutic insufficiency occurs when treatment is not adequate or sufficient. Therapeutic failure implies the persistence of signs and symptoms of leprosy even after 12 to 24 doses of treatment or persistence of intact bacilli.^4^ Finally, reinfection corresponds to re-exposure to the bacillus with clinical disease after discharge due to cure.^5^

The authors aimed to characterize patients with leprosy treated in a state health reference service in southeastern Brazil between 2010 and 2020, classifying them according to the criteria of recurrence, therapeutic insufficiency, therapeutic failure and reinfection, through an observational, non-interventionist retrospective study, carried out based on a review of medical records and after approval by the ethics committee of that institution (CAAE number 36518420.0.0000.5404).

With an average of 30 to 50 patients attending the unit per month, eighteen patients were found who underwent retreatment for leprosy during the period (Table 1, Table 2).

**Table 1:** Epidemiological profile of leprosy patients undergoing retreatment from 2010 to 2020.

Variable	Patients (n = 18)
Gender	7 female
	11 male

Level of schooling	10 Elementary School
	6 High School/Higher Education
	2 unknown

Age at 1^st^ treatment (mean)	38.9 years (15 to 75 years)

Clinical form at first treatment	
TL	1
BL	10
LL	7

Bacilloscopic index (BI) at 1^st^ treatment	
≤ 3	7
> 3	9
Unknown	2

Degree of disability at 1^st^ treatment	
0	1
1	13
2	2
Unknown	2

Number of treatments	
2 treatments	14
≥ 3 treatments	4

Time between 1^st^ and 2^nd^ treatments (mean)	6 years (2 to 20 years)

TL, Tuberculoid Leprosy; BL, Borderline Leprosy; LL, Lepromatous Leprosy.

Source: Direct search.

**Table 2:** Classification of leprosy retreatment cases according to the Ministry of Health criteria.

Variable	Patients (n = 18)
Recurrence	3
Therapeutic insufficiency	
High bacillary load (BI ≥ 3+)	9
Misclassification	1
Treatment regimen	1
Therapeutic failure	2
Reinfection^a^	2

BI, Bacilloscopic Index.

Source: Direct search.

a Interval between the first and last treatment greater than 20 years and positive epidemiology.

In eight patients (44%) bacterial resistance was tested using molecular biology, with only one showing resistance to dapsone (mutation in the *folP1* gene), classified during retreatment as therapeutic failure. All patients developed a leprosy reaction between treatments (83.3% type 2). Six patients were treated with multibacillary multidrug therapy (MB-MDT) without dapsone (with ofloxacin as a replacement drug) in the first treatment and ten used the standard MB-MDT regimen for 12 months.

The therapeutic regimen most often used in retreatment was the standard MB-MDT, with an extension of 24 months, but, in selected cases, minocycline and ofloxacin were additionally used.

The described data are in agreement with the literature, which states that leprosy recurrence is more common in multibacillary patients (85%) with time to retreatment exceeding five years.^6^ However, Brito et al.^7^ described reaction episodes in only 34% of the treated patients, in contrast to the high frequency found in the present study. Leprosy reactions, especially if chronic or ongoing, can raise many doubts regarding the presence of active disease.^8^ The persistence of the leprosy reaction for a period equal to or greater than five years in patients with a high bacillary load, as well as the presence of viable bacilli (or forming globi) on histopathology were criteria used in the present study to indicate retreatment.

Diagnoses of recurrence and bacterial resistance in leprosy have been a growing global concern. Between 2017 and 2021, southeastern Brazil was the third region to document most cases of recurrence in the country.^2^

Drug resistance is one cause of therapeutic failure. In Brazil, there is a frequency of around 10% of resistance to dapsone in recurrence cases, similar to what has been described in the rest of the world.^9^, ^10^ Recent efforts have been made to better investigate cases of recurrence, with the inclusion of drug resistance and mutation research at a national level.^9^, ^10^

More than half of the cases reviewed in the present study were reclassified as therapeutic insufficiency. These cases had a high Bacilloscopic index (BI), a risk factor for the persistence of bacilli when treated with the conventional MB-MDT 12 doses regimen.^9^, ^10^ It is also noteworthy the finding of five patients (27.8%) who were treated with replacement regimens in the first treatment (ofloxacin instead of dapsone), which may raise doubts about the real effectiveness of alternative medications.^6^

Therapeutic insufficiency is a frequent cause for retreatment in leprosy. A better definition of the criteria that classify retreated patients is necessary. Systematic research on drug resistance and the development of new and better therapeutic strategies can help reduce the incidence of the disease in Brazil.

## Financial support

None declared.

## Authors’ contributions

Guilherme Holanda Bezerra: Design of the study; analysis of results; data collection; drafting and editing of the manuscript; review and approval of the final version of the manuscript.

Paulo Eduardo Neves Ferreira Velho: Design of the study; analysis of results; drafting and editing of the manuscript; review and approval of the final version of the manuscript.

Andréa Fernandes Eloy da Costa França: Design of the study; analysis of results; data collection; drafting and editing of the manuscript; review and approval of the final version of the manuscript.

## Conflicts of interest

None declared.
